# Comparison of [68Ga]-FAPI PET/CT and [18F]-FDG PET/CT in Multiple Myeloma: Clinical Experience

**DOI:** 10.3390/tomography8010024

**Published:** 2022-02-01

**Authors:** Umut Elboga, Ertan Sahin, Yusuf Burak Cayirli, Merve Okuyan, Gokmen Aktas, Handan Haydaroglu Sahin, Ilkay Dogan, Tulay Kus, Dervis Murat Akkurd, Ufuk Cimen, Vuslat Mumcu, Benan Kilbas, Yusuf Zeki Celen

**Affiliations:** 1 Department of Nuclear Medicine, Gaziantep University, Gaziantep 27310, Turkey; ertansah@gantep.edu.tr (E.S.); yusufburakcayirli@gantep.edu.tr (Y.B.C.); merveokuyan@gantep.edu.tr (M.O.); ufuk.cimen1992@hotmail.com (U.C.); vuslatmumcu@hotmail.com (V.M.); celen@gantep.edu.tr (Y.Z.C.); 2 Department of Oncology, Medical Park Private Hospital, Gaziantep 27090, Turkey; gokmen.aktas@medicalpark.com.tr; 3 Department of Hematology, Gaziantep University, Gaziantep 27310, Turkey; handansahin@gantep.edu.tr (H.H.S.); dervismuratakkurd@gantep.edu.tr (D.M.A.); 4 Department of Biostatistics, Gaziantep University, Gaziantep 27310, Turkey; ilkaydogan@gantep.edu.tr; 5 Department of Oncology, Gaziantep University, Gaziantep 27310, Turkey; tulaykus@gantep.edu.tr; 6 Department of R&D, Moltek Health Services Production & Marketing Inc., Kocaeli 41400, Turkey; benan.kilbas@moltek.com.tr

**Keywords:** multiple myeloma, [68Ga]FAPI, [18F]FDG

## Abstract

Objective: In this study, we aimed to compare [68Ga]FAPI PET/CT and [18F]FDG PET/CT imaging to detect lesions in multiple myeloma. Methods: A total of 14 patients with multiple myeloma who underwent [68Ga]FAPI PET/CT and [18F]FDG PET/CT imaging were included in this retrospective study. SUV_max_ values of [68Ga]FAPI and [18F]FDG were compared according to lesion locations. Also, lesion localization ability of both imaging methods was compared on the patient basis. Results: In 4 of 14 patients, [68Ga]FAPI PET/CT and [18F]FDG PET/CT have not detected any bone lesions. In 8 of the remaining 10 patients [18F]FDG PET/CT detected bone lesions but in this group, 6 patients showed more higher SUV_max_ values than [18F]FDG PET/CT in [68Ga]FAPI PET/CT.In contrast, 2 of 8 patients showed more higher SUV_max_ values than [68Ga]FAPI PET/CT in [18F]FDG PET/CT. Moreover, [68Ga]FAPI PET/CT detected bone lesions in two patients, which werenot detected by [18F]FDG PET/CT. Also, in five patients, [68Ga]FAPI PET/CT showed more bone lesions in comparison with[18F]FDG PET/CT. Only one patient, [18F]FDG PET/CT showed more bone lesions. Three extramedullary involvements were observed in the following locations: lung, presacral lymph node, and soft tissue mass lateral to the right maxillary sinus. Among these involvements, higher SUV_max_ values were observed in the lung and presacral lymph node with [68Ga]FAPI compared to [18F]FDG. However, the soft tissue mass showed a higher SUV_max_ value in [18F]FDG than [68Ga]FAPI. Conclusions: No significant superiority was observed in [68Ga]FAPI PET/CT over [18F]FDG PET/CT in patients with MM. However, [68Ga]FAPI PET/CT can be utilized as a complementary imaging method to [18F]FDG PET/CT in some settings, especially in low-[18F]FDG affinity and inconclusive cases. Considering the favorable aspects of [68Ga]FAPI PET/CT in MM, such as low background activity, absence of non-specific bone marrow, and physiological brain involvement, further studies with a larger sample size should be conducted.

## 1. Introduction

Multiple myeloma (MM) is a neoplastic disease of the bone characterized by uncontrolled clonal proliferation of plasma cells. Bone disease, one of the major causes of mortality and morbidity in MM, occurs at the time of diagnosis in approximately two-thirds of patients and during disease in almost all patients [1,2]. For this reason, imaging plays a very important role in the diagnosis of MM, which ismainly based on bone involvement. First, detection of osteolytic bone lesions and end-organ damage closely associated with the disease is essential to determine the need for immediate treatment [3]. Unlike other malignancies that metastasize to the bone, bone lesions in MM do not cause new bone formation as they are lytic in nature [4]. Skeletal lesions are seen in the spine, pelvis, skull, ribs, sternum, and proximal appendicular skeleton, in order of frequency [5]. Skeletal examinations have been replaced by whole body CT (computed tomography), whole body MRI (magnetic resonance imaging), or [18F]FDG PET/CT (18F-fluoro-deoxy-glucose positron emission tomography) in routine studies to identify lesions that define MM. Both MRI and [18F]FDG PET/CT have the advantage of evaluating bone marrow-occupying lesions before bone resorption is seen on CT [6]. The European Myeloma Network and European Society for Medical Oncology guidelines have recommended whole body CT as the imaging modality of choice for the initial assessment of MM-related lytic bone lesions and MRI is the gold standard imaging modality for detecting bone marrow involvement. However, [18F]FDG PET/CT provides valuable prognostic data and is preferred for evaluating response to treatment. Previous studies have shown that [18F]FDG PET/CT aid is useful in detecting both osseous and extra osseous MM related lesions [7,8,9]. On the other hand, low hexokinase-2 expression in MM may cause false negative results [10]. Furthermore, recent steroid treatment, small lytic lesions in the skull close to the brain, and hyperglycemia are other possible causes of false negative results on [18F]FDG PET/CT [11]. Although [18F]FDG-avid lesions and extramedullary involvement of MM are associated with a poor prognosis, possible false negative results, as mentioned above, may have a negative clinical impact on the initial assessment of MM [12].

Fibroblast activation protein (FAP), a member of the dipeptidyl peptidase IV (DPP-IV) family, is expressed on the surface of cancer-associated fibroblasts (CAF). Therefore, FAP expression is subjected to both diagnostic and therapeutic studies [13,14]. Studies on FAP have drawn attention to increased expression in various cancers. Although FAP expression is high in cancer stroma, it is considerably low in normal adult tissues, except for sites of active tissue damage, chronic inflammation, and remodeling [15]. The relatively specific expression of FAP in the tumor microenvironment has made it possible to develop FAP inhibitors (FAPIs) [16,17]. Subsequently, [68Ga]-labeled FAPI provided PET/CT images with high tumor-to-background ratios (TBRs) in a wide variety of cancer patients, suggesting high potential for FAP-targeted diagnosis and possibly targeted, radioligand therapies in the future. Considering that [68Ga]FAPI PET/CT demonstrates low background activity, including bones, it may be beneficial in MM lesions with low [18F]FDG affinity [18,19].

In this study, we aim to compare [68Ga]FAPI PET/CT and [18F]FDG PET/CT in terms of bone or extramedullary involvement in multiple myeloma.

## 2. Materials and Methods

### 2.1. Patients

This retrospective study was approved by the Clinical Research Ethics Committee of our university and conducted in accordance with the 1964 Helsinki declaration for ethical standards. For cases with low [18F]FDG affinity, [68Ga]FAPI PET/CT was applied after [18F]FDG PET/CT enrolled in this study between September 2020 and February 2021, within the scope of the local institutional license for the magistral production and use of [68Ga]-labeled radiopharmaceuticals, and the use of experimental radiopharmaceuticals in determined patient groups retrospectively scanned from the medical record archive. Inclusion criteria were (1) being older than 18 years, (2) having histopathological confirmation of MM, and (3) being able to provide informed consent. Patients’ exclusion criteria were (1) pregnancy or lactation; (2) inability or unwillingness to provide written informed consent;and (3) having arthritis, chronic inflammatory condition, or cirrhosis. Furthermore, levels of LDH (lactate dehydrogenase), CRP (C-reactive protein), and beta-2 microglobulin were obtained alongside histopathological features and immunoglobulin secretion situation in all patients.

### 2.2. Staging in Multiple Myeloma: International Staging System (ISS) and Durie and Salmon PLUS Staging System

Beta-2-microglobulin levels are divided into three stages according to ISS criteria [20,21,22]. Accordingly, patients with β_2_-microglobulin levels less than 3.5 mg/L were classified as stage I, patients with serum β_2_-microglobulin levels equal to or higher than 3.5 mg/L and less than 5.5 mg/L were classified as stage II, and patients with a serum β_2_-microglobulin level equal to or higher than 5.5 mg/L were stage III [22].

The Salmon–Durie classification of MM is based on three stages and additional subclassifications. In stage I, the MM cell mass is less than 0.6 × 10^12^ cells/m^2^, and all the following are present: hemoglobin value >10 g/dL, serum calcium value < 12 mg/dL (normal), normal bone structure (scale 0) or only a solitary bone plasmacytoma on radiographs, low M-component production rates (IgG value < 5 g/dL, IgA value < 3 g/dL, urine light-chain M component on electrophoresis < 4 g/24 h). In stage II, the MM cell mass is 0.6–1.2 × 10^12^ cells/m^2^ or more. The other values fit neither those of stage I nor those of stage III. In stage III, the MM cell mass is >1.2 × 10^12^ cells/m^2^, and all of the following are present: hemoglobin value < 8.5 g/dL, serum calcium value >12 mg/dL, advanced lytic bone lesions (scale 3) on radiographs, high M-component production rates (IgG value greater than 7 g/dL, IgA value greater than 5 g/dL, urine light-chain M component on electrophoresis greater than 12 g/24 h). Integration of imaging the MRI or PET findings were used to stage the disease in each patient according to the Durie and Salmon PLUS staging system: stage I (0–4 lesions), stage II (5–20 lesions), and stage III (>20 lesions) [23,24,25].

### 2.3. [68. Ga]FAPI-04

FAPI-04 was obtained from MedChem Express LLC. The pharmaceutical grade (68Ge)/(68Ga) generator (50 mCi) and disposable cassettes were supplied by Eckert and Ziegler Eurotope GmbH. Purification cartridge CM (Sep-Pak AccellPlus CM Plus Light Cartridge, 130 mg Sorbent per Cartridge, 37–55 µm, WAT023531) was well-established in the cassette accessories. Other chemicals and materials were purchased from Aldrich (Chemical Company Inc., Istanbul, Turkey) in ultra-pure and trace metal basis grades. The HPLC analyses were performed by Modular-Lab HPLC (Eckert and Ziegler Inc., Wilmington, DC, USA) device using ACE-3 C18 150 × 3.0 mm^2^ column.

### 2.4. Radiolabeling Procedure

The radiolabeling process was performed by a fully automated system without any manual interaction. (68Ga)^3+^ was eluted with 0.1 N HCl solution (8.0 mL) followed by passing through the pre-concentration on a strong cation exchange (SCX) cartridge. The (68Ga) activity was recovered from the SCX cartridge by 0.9 mL eluent (5 M NaCl/HCl (0.1 M)). The reaction vial is filled by 2 mL of H^2^O, 0.4 mL of sodium acetate buffer (pH is around 4.5), 0.2 mL of ethanol, and 50 µg of FAPI-04. Then, (68Ga) activity was transferred to the reaction vial, and it was heated to 95 °C for 10 min. After the completion of the reaction, the reaction medium was cooled down and crude product was diluted by adding 5.0 mL of 0.9% NaCl and subsequently purified by the CM cartridge. Finally, the reaction mixture was passed through a millipore filter (0.22 µm) and was injected intravenously after more than a 98% radiochemical purity with 88% radiochemical yield. The radiochemical purity was analyzed by R-HPLC and free (68Ga) was detected at RT = 2.2 min, whereas [68Ga]FAPI-04 was detected at RT = 3.99 min. (ACE-3 C18 150 × 3.0 mm^2^ column, isocratic flow 0.6 mL/min; mobile phase: 85% H^2^O (0.1 TFA) and 15% ACN (0.1 TFA).

### 2.5. PET/CT Protocol and Image Analysis

All patients were examined using a PET/CT system (Discovery™ IQ; GE Healthcare, Milwaukee, Brookfield, WI, USA) combining a dedicated, five-ring PET scanner with Light Burst Technology Inc. (London, UK). All patients had fasted for at least 6 h before [18F]FDG administration. Blood glucose was tested to ensure a normal blood glucose level. A whole-body (from top of head to mid-thigh) PET/CT was performed approximately 60 min after the intravenous injection of [18F]FDG (3.7–5.4 MBq/kg, 0.10–0.15 mCi/kg) according to the clinical standard protocol for tumor imaging. All patients underwent whole-body [68Ga]FAPI PET/CT within one week, with no specific preparation required before [68Ga]FAPI administration. The PET/CT scan was performed after the intravenous injection of [68Ga]FAPI (1.85–3.7 MBq/kg, 0.05–0.1 mCi/kg).

PET imaging was performed for 60 min for [18F]FDG and 30 min for [68Ga]FAPI (5–6mCi) after injection, with 5 bed positions of 3 min each. Emission PET data were acquired from the base of the skull to the upper thigh in 3D mode using a Discovery ST scanner (Discovery™ IQ; GE Healthcare), and then they were reconstructed with non-contrast CT (tube rotation time 1s/revolution, 120 kV, 60 mA, 7.5 mm/rotation, scan length 867 mm) by iterative reconstruction (ordered-subsets expectation maximization with 2 iterations and 30 subsets, field of view = 600 mm, slice thickness = 3.27 mm).

PET, CT, and fused whole-body images displayed in axial, coronal, and sagittal planes were available for review. A semi-quantitative analysis of tracer activity was measured as the maximal standardized uptake value (SUV_max_) of [18F]FDG or [68Ga]FAPI using the provided software (AW VolumeShare, GE Healthcare). [18F]FDG PET/CT and [68Ga]FAPI PET/CT images were evaluated both qualitatively and semi-quantitatively. For the semi-quantitative analysis, polygonal regions of interest (ROIs) were first drawn on CT images and then copied to attenuation-corrected PET images using the Advantage Workstation (version 4.4, GE Healthcare). For tumors with a hypermetabolic lesion, ROIs were placed at every transaxial plane of CT images that contained the hypermetabolic lesion. Meanwhile, for those without visually discernible [18F]FDG or [68Ga]FAPI uptake, ROIs were drawn to cover the whole tumor. In cases of multiple malignant nodules, an ROI was drawn on the largest one. Maximum standardized uptake value (SUV_max_) was calculated with the injected dose and the patient’s body weight. Any non-physiological uptake greater than background blood-pool activity or adjacent normal tissue background on [18F]FDG or [68Ga]FAPI PET was included in the study. Positive findings on PET were localized to anatomic images from the non-enhanced CT. The PET/CT findings were grouped as intramedullary bone lesions, lymph nodal metastasis, and distant metastasis. The lesion number and SUV_max_ of the lesion with the highest pathological tracer accumulation were recorded for each bone lesion, lymph node, or distant metastasis site for both [18F]FDG and [68Ga]FAPI-04 PET/CT.

The PET/CT images were carefully evaluated by two experienced nuclear medicine physicians. Images were reviewed independently of the scans.

### 2.6. Reference Standard

Patients had undergone comprehensive re-evaluation, including clinical and hematological data, as well as the appraisal of bone lesions on an ultimate MRI. The histological specimen, hematological parameters (serum levels of beta-2-microglobulin (B_2_M), C-reactive protein (CRP) and lactate dehydrogenase (LDH), bone marrow aspirate (biopsy to determine infiltration by plasma cells), monoclonal proteins (M-proteins) in the serum or urine, serum-free light chain (FLC) ratio, and IgG isotype were also taken as the reference standard. Any performed therapeutic regimen wasregistered from the patients’ medical record. Patients whose lesions were previously treated by external radiotherapy before PET/CT imaging were excluded from this study to avoid confounding items.

### 2.7. Statistical Analysis

We performed descriptive analyses for the characteristics of patients. The Bland–Altman analysis was used to evaluate the correlation between SUV_max_values of [68Ga]FAPI and [18F]FDG. Also, *p*-values of <0.05 were defined as statistically significant. All statistical analyses were performed using IBM SPSS Statistics Version 26 for Mac v15.41.

## 3. Results

The study was conducted prospectively in 14 patients with MM. 50% of the patients were female and 50% were male. A comprehensive study was performed to demonstrate the following myeloma-related parameters in all eligible patients: serum M-protein; full immunoglobulin and free light chain type; serum levels of beta-2-microglobulin (B2M), C reactive protein (CRP) and lactate dehydrogenase (LDH); bone marrow aspirate and biopsy to determine infiltration by plasma cells. Patients were staged according to the ISS and Durie Salmon PLUS staging system. The baseline characteristics of the patients were demonstrated in Table 1.

In 4 of 14 patients, [68Ga]FAPI PET/CT and [18F]FDG PET/CT did not detect any bone lesions. In 8 of the remaining 10 patients, [18F]FDG PET/CT detected bone lesions, but in this group, 6 patients showed a higher SUV_max_ value than [18F]FDG PET/CT in [68Ga]FAPI PET/CT. In contrast, 2 of 8 patients showed a higher SUV_max_ value than [68Ga]FAPI PET/CT in [18F]FDG PET/CT. Moreover, [68Ga]FAPI PET/CT detected bone lesions in two patients which werenot detected by [18F]FDG PET/CT. Also, in five patients, [68Ga]FAPI PET/CT showed more bone lesions in comparison with [18F]FDG PET/CT. Only in one patient [18F]FDG PET/CT show more bone lesions (Table 2).

Mean ± standard deviation (SD) of SUV_max_ values of bone lesions in [18F]FDG and [68Ga]FAPI were 6.15 ± 3.97 (*n* = 8) and 9.30 ± 3.22 (*n* = 10), respectively. There was no statistically significant difference between these imaging modalities in terms of SUV_max_ values of bone lesions (*p* = 0.081).

**Figure 1:**
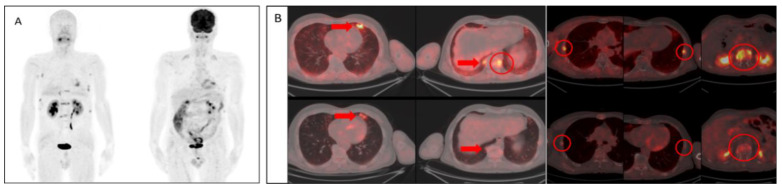
Patient No 1. 58 year-old-male diagnosed with MM. (**A**) Visual comparison of maximum intensity projections of both modalities (Left [68Ga]FAPI, right [18F]FDG) showed superior image quality and higher specific activity retentions with [68Ga]FAPI PET/CT. (**B**) In axial plane evaluations, both imaging methods demonstrated extramedullary involvement of MM in the lungs (Arrows); however, SUV_max_ values of the lesions were noted to be higher in [68Ga]FAPI PET/CT (Upper row). Also, significantly higher activity uptake was observed in the bone lesions (Circles) with [68Ga]FAPI (Upper row) when compared to [18F]FDG (Lower row).

**Figure 2:**
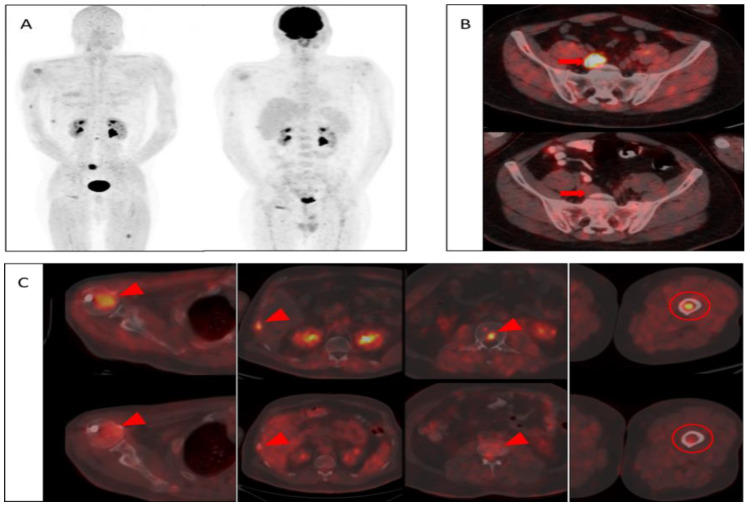
Patient No 3. 39-year-old male diagnosed with MM. (**A**) Visual comparison of maximum intensity projections of both modalities showed superior image quality and higher specific activity retentions in [68Ga]FAPI PET/CT (Left) without non-specific diffuse medullary uptake as in [18F]FDG PET/CT (Right). (**B**) In axial plane evaluations, both imaging methods demonstrated extramedullary involvement of MM in the presacral lymph node (Arrows); however, SUV_max_ value of the lymph node were noted to be significantly higher in [68Ga]FAPI PET/CT (Upper row). (**C**) In the visual evaluation of the bone lesions (Arrow heads), [68Ga]FAPI PET/CT (Upper row) demonstrated higher activity retentions in all locations with significantly lower background activity compared to [18F]FDG PET/CT (Lower row). Also, more pronounced focal activity retention was observed in the intramedullary region of the left femur with [68Ga]FAPI PET/CT (Upper row) compared to [18F]FDG PET/CT (Lower row), without morphological pathology (Circles).

Both PET/CT imaging modalities demonstrated extramedullary involvement in the following locations in three patients: lung, presacral lymph node, and soft tissue mass lateral to the right maxillary sinus. Among these involvements, higher SUV_max_ values were observed in the lung (Figure 1B) and presacral lymph node (Figure 2B) with [68Ga]FAPI compared to [18F]FDG. However, the soft tissue mass (indicated in Table 1) showed a higher SUV_max_ value in [18F]FDG than [68Ga]FAPI (Table 2).

## 4. Discussion

Imaging modalities in MM play an important role in diagnosis, determining disease dissemination, and evaluation of response to treatment. On whole-body x-radiography (WBXR), multiple myeloma lesions typically have a perforated osteolytic appearance. However, for it to be detected as a lytic lesion on radiography, at least 50% of the relevant trabecular bone must be destroyed. On the other hand, computed tomography (CT) has been found to be more sensitive than WBXR in detecting lytic lesions with less than 5% trabecular bone destruction [5,20,21]. Both WBXR and CT assess the damage of tumor cells to mineralized bone tissue, but do not provide information on tumor cell viability or activity. On the other hand, magnetic resonance imaging (MRI) and PET/CT are more functional imaging methods, as they can evaluate the diffusion of interstitial water molecules and glucose uptake, which are markers of viability in tumor tissue [22].The International Myeloma Working Group has recommended that use of [18F]FDG PET/CT in MM could provide beneficial information about the metabolism of plasma cells. The most important advantage of [18F]FDG PET/CT is its ability to assess disease burden with highaccuracy and to distinguish metabolic activity among various lesions [23,26]. However, utilization of [18F]FDG PET/CT in patients with MM has some limitations. Studies have shown that false positive results can be detected with [18F]FDG PET/CT in certain situations, such as low [18F]FDG affinity, recent steroid treatment, hyperglycemia, non-specific bone marrow involvements, and small lytic lesions in the skull close to the brain [10,11]. High [18F]FDG affinity associated with poor prognosis and possible false negative results may have a negative clinical impact on the initial assessment of MM [12]. Therefore, novel imaging modalities, such as [68Ga]FAPI PET/CT, may be useful.

**Figure 3:**
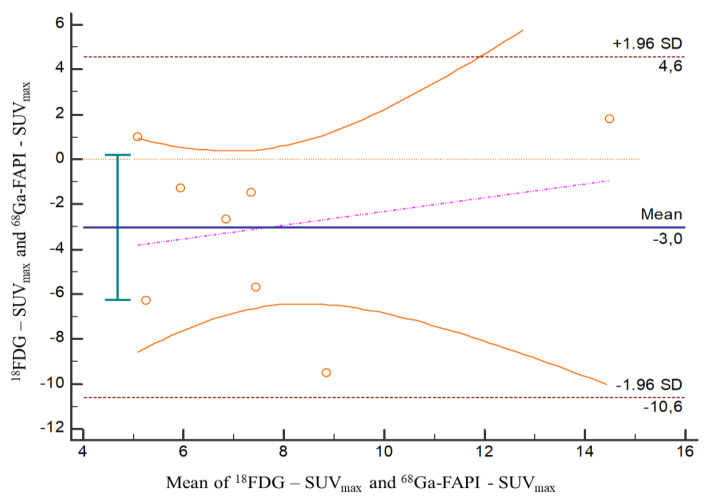
Bland–Altman plot for comparison between SUV_max_ values of bone lesions detected in [18F]FDG PET/CT and [68Ga]FAPI PET/CT. Mean SUV_max_ value was 6.15 ± 3.97 (*n* =8) in [18F]FDG and 9.30 ± 3.22 (*n* =10) in [68Ga]FAPI. There was no statistically significant difference between SUV_max_ values of bone lesions detected in both modalities (*p* = 0.081).

In the current study, [68Ga]FAPI PET/CT showed high quality images with specific tumor activity. Also, there were no non-specific involvements as well as favorable low background activity in visual evaluations in agreement with the literature [18,19,27,28,29]. In two of the patients, [68Ga]FAPI PET/CT showed multiple bone lesions with considerably higher SUV_max_ values, while [18F]FDG PET/CT have not shown hyper-metabolic lesions. Also, more bone lesions were detected in five of the patients with [68Ga]FAPI PET/CT. In six of the patients, both modalities showed similar results, and in one of the patients, more foci were identified in [18F]FDG PET/CT when compared to [68Ga]FAPI PET/CT. Also in a patient, it is noteworthy that [68Ga]FAPI PET/CT showed focal intramedullary bone involvement without morphological pathology, whereas [18F]FDG PET/CT showed no significant uptake (Figure 2C). However, there was no statistically significant difference between these imaging modalities in terms of SUV_max_ values of bone lesions (Figure 3).

Both imaging methods showed extramedullary involvement in three patients with different activity characteristics. Extramedullary involvement of two patients showed higher [68Ga]FAPI uptake than [18F]FDG, however more pronounced [18F]FDG retention was observed in the other patient.

In a recent study, lesion-based comparison of [68Ga]FAPI PET/CT and [18F]FDG PET/CT in three patients with MM demonstrated that [18F]FDG had greater sensitivity than [68Ga]FAPI. Furthermore, [18F]FDG PET/CT showed significantly higher SUV_max_ values compared to [68Ga]FAPI PET/CT [29]. In contrast, no statistically significant association was found between the SUV_max_ values of both imaging methods in the current study.

In addition to the relatively small sample size, our study may have limitations as it did not include histopathological confirmation of all metastatic lesions, except primary lesions and target lesions.

## 5. Conclusions

In conclusion, no significant superiority was observed in [68Ga]FAPI PET/CT over [18F]FDG PET/CT in patients with MM in this study. The results of our study did not make a significant contribution to changing treatment management in the patients. However, [68Ga]FAPI PET/CT can be utilized as a complementary imaging method to [18F]FDG PET/CT in some settings, especially in low [18F]FDG affinity and inconclusive cases. Considering the favorable aspects of [68Ga]FAPI PET/CT in MM, such as low background activity, absence of non-specific bone marrow, and physiological brain involvement, further studies with a larger sample size should be conducted.

## Figures and Tables

**Table 1:** Baseline characteristics of patients.

No	A/G	Plasma Cell Percentage	Diagnostic Localization	Subtype of Ig (or Non-Secretory Type)	Type of Light Chain	ISS	LDH	CRP	B_2_ Microglobulin	Durie Salmon Staging
1	58/m	80	Bone marrow	IgA	Lambda	1	215	6.5	2.8	3
2	64/f	80	Bone marrow	Non-secretory type	Lambda	3	234	2.2	14.5	3
3	39/m	80	Plasmocitoma	IgG	Kappa	1	243	2.7	3.09	3
4	65/f	70	Bone marrow	IgG	Lambda	3	157	2.5	14.3	2
5	40/f	40	Plasmocitoma	IgG	Kappa	1	139	2.5	2.2	2
6	58/m	70	Bone marrow	IgG	Lambda	1	212	11.6	3.2	3
7	81/m	50	Bone marrow	IgG	Kappa	3	1366	23.7	9.2	3
8	59/f	80	Plasmocitoma	IgG	Kappa	2	185	77.2	3.9	3
9	54/m	50	Bone marrow	Non-secretory type	Lambda	1	159	14	3.1	2
10	55/f	40	Bone marrow	IgA	Lambda	1	186	6.7	1.6	1
11	57/f	40	Plasmocitoma	IgG	Kappa	2	146	1.6	2.7	3
12	69/f	15	Bone marrow	IgG	Lambda	1	438	1.5	3	3
13	58/m	24	Bone marrow	IgG	Lambda	1	159	77	3.1	3
14	66/m	50	Bone marrow	IgA	Lambda	3	125	1	8.3	3

No: Patient’s number; A/G: Age/Gender; m: Male, f: Female; LDH: Lactate dehydrogenase; CRP: C-reactive protein; B2M: Beta-2-microglobulin; ISS: International Staging System.

**Table 2:** Number and SUV_max_ values of lesions detected by [18F]FDG PET/CT and [68Ga]FAPI PET/CT in bone and extramedullary involvement.

No	Number of Bone Lesions with FDG	FDG SUV_max_ Value	Number of Bone Lesions with FAPI	FAPI SUV_max_ Value	Extramedullary Involvement ^a^	Extramedullary Involvement ^b^
1	1	4.1	10	13.6	8.1^1^	13^1^
2	0	0	6	6.5		
3	4	5.3	13	6.6	2.7^2^	14.7^2^
4	0	0	0	0		
5	2	2.1	2	8.4		
6	0	0	8	13.1		
7	0	0	0	0		
8	6	5.6	7	4.6	11.7^3^	5.7^3^
9	3	15.4	3	13.6		
10	0	0	0	0		
11	14	6.6	4	8.1		
12	0	0	0	0		
13	6	5.5	9	8.2		
14	2	4.6	4	10.3		

^a^ Metastatic region and FDG SUV_max_. ^b^ Metastatic region and FAPI SUV_max_. ^1^ Lung. ^2^ Presacral lymph node. ^3^ Soft tissue mass in the lateral neighborhood of the right maxillary sinus.

## Data Availability

All data generated or analyzed during this study are included in this article [and/or] its supplementary material files. Further enquiries can be directed to the corresponding author.
